# The Genome of *Ganderma lucidum* Provide Insights into Triterpense Biosynthesis and Wood Degradation

**DOI:** 10.1371/journal.pone.0036146

**Published:** 2012-05-02

**Authors:** Dongbo Liu, Jing Gong, Wenkui Dai, Xincong Kang, Zhuo Huang, Hong-Mei Zhang, Wei Liu, Le Liu, Junping Ma, Zhilan Xia, Yuxin Chen, Yuewen Chen, Depeng Wang, Peixiang Ni, An-Yuan Guo, Xingyao Xiong

**Affiliations:** 1 Hunan Agricultural University, Changsha, Hunan, China; 2 State Key Laboratory of Sub-health Intervention Technology, Changsha, Hunan, China; 3 Hubei Bioinformatics and Molecular Imaging Key Laboratory, College of Life Science and Technology, Huazhong University of Science and Technology, Wuhan, Hubei, China; 4 Beijing Genome Institute (BGI-Shenzhen), Shenzhen, Guangdong, China; 5 Hunan Wanyuan Bio-tech Co., Ltd., Changsha, Hunan, China; 6 Nextomics Biosciences Co., Ltd., Wuhan, Hubei, China; 7 School of Bioscience and Bioengineering, South China University of Technology, Guangzhou, Guangdong, China; 8 Key Laboratory for Crop Germplasm Innovation and Utilization of Hunan Province, Hunan Agricultural University, Changsha, Hunan, China; Hospital for Sick Children, Canada

## Abstract

**Background:**

*Ganoderma lucidum* (Reishi or Ling Zhi) is one of the most famous Traditional Chinese Medicines and has been widely used in the treatment of various human diseases in Asia countries. It is also a fungus with strong wood degradation ability with potential in bioenergy production. However, genes, pathways and mechanisms of these functions are still unknown.

**Methodology/Principal Findings:**

The genome of *G. lucidum* was sequenced and assembled into a 39.9 megabases (Mb) draft genome, which encoded 12,080 protein-coding genes and ∼83% of them were similar to public sequences. We performed comprehensive annotation for *G. lucidum* genes and made comparisons with genes in other fungi genomes. Genes in the biosynthesis of the main *G. lucidum* active ingredients, ganoderic acids (GAs), were characterized. Among the GAs synthases, we identified a fusion gene, the N and C terminal of which are homologous to two different enzymes. Moreover, the fusion gene was only found in basidiomycetes. As a white rot fungus with wood degradation ability, abundant carbohydrate-active enzymes and ligninolytic enzymes were identified in the *G. lucidum* genome and were compared with other fungi.

**Conclusions/Significance:**

The genome sequence and well annotation of *G. lucidum* will provide new insights in function analyses including its medicinal mechanism. The characterization of genes in the triterpene biosynthesis and wood degradation will facilitate bio-engineering research in the production of its active ingredients and bioenergy.

## Introduction

*Ganoderma lucidum* (Leyss. ex. Fr) Karst., Ling-Zhi in Chinese and Reishi in Japanese, belonging to the Ganodermataceae of Aphyllophorales in Basidiomycetes [1], is a widely distributed fungus in the tropic and subtropics of Asia, Africa and America [2], with the most diversities in China. *G. lucidum* is one of the most famous Traditional Chinese Medicines and has been widely used as a tonic for longevity and overall health in China for thousands of years [3]. *G. lucidum* has been proved with remarkable pharmacological activities and therapeutic effects in immuno-modulation, anti-cancer, anti-radiation and detoxification for various human diseases [4]–[7]. However, the accumulation of its active ingredients and the pharmacological mechanisms are mainly unknown. The genome sequence and gene annotation of *G. lucidum* will provide key resources and may speed up the function research of *G. lucidum* to human health.

Ganoderic acids (GAs), one of the main active ingredients of *G. lucidum*, are a kind of triterpenoid secondary metabolites and shown the ability to participate in many biological activities including antitumor, antioxidant, etc. [8]. However, the content of GAs is very low and is suggested to be the quality indicator of *G. lucidum* in Japan [1], [9]. It is suggested that the triterpene backbone of GAs could be biosynthesized via the mevalonic acid (MVA) pathway. Several genes in this pathway have been cloned in *G. lucidum*, including 3-Hydroxy- 3- methylglutaryl- CoA reductase (HMGR) [10], Farnesyl diphosphate synthase (FPPs) [11], Squalene synthase (SQS) [12], and Lanosterol synthase (also namely 2, 3-oxidosqualene lanosterol cyclase, OSC) [13]. However, it rarely reported about the processes of decoration after the triterpene backbone biosynthesis, such as cyclization and glycosylation, which are very important for GAs synthesis. The genome sequence is expected to characterize the enzymes of these key steps in the GAs biosynthesis.

*G. lucidum* is one of the white-rot fungi that grow on the dead trees by degrading cellulose, hemicellulose and lignin. Lignin, one of the main polymeric components of plant cell wall, is highly resistant to chemical and biological degradation [14]. Although there are some reports mentioned ligninolytic enzymes, the mechanism of lignin degradation is still not fully understood [14]–[16]. In addition, different enzymatic systems are employed in different fungi [17]. As one of the dominant organisms decomposing lignocellulose, it would be interesting to figure out the enzymatic system and genes of *G. lucidum* in wood degradation.

With the development of next-generation DNA sequencing, several macrofungi have been sequenced and analyzed to illuminate different aspects. Ohm *et al.* [18] studied the fruiting bodies formation and lignocelluloses degradation of *Schizophyllum commune*. Stajich *et al.* [19] completed the chromosome assembly of *Coprinopsis cinerea*, and investigated the meiotic recombination, genes and gene families and so on. Martin *et al.* illustrated the different ways of genetic predisposition for symbiosis in basidiomycete *Laccaria bicolor* [20] and ascomycete *Tuber melanosporum* Vittad. [21]. With these fungi genomes, it is possible to make full annotation and comparison for *G. lucidum* genomes. The genome annotation of *G. lucidum* will provide important data to further function and mechanism research in *G. lucidum* and comparative genomics in fungi.

In this study, we sequenced the genome of monokaryotic *G. lucidum* strain isolated from China and assembled a 39.9 Mb genome. We made full annotations with the predicted genes in this genome and compared them with other fungi genomes. With integrated gene prediction and annotation, we illuminated the synthesis of GAs as a model system to study triterpenoid biosynthesis in fungi. Besides the importance of understanding the biosynthesis of this active ingredient, insights into the enzyme systems of lignocelluloses degradation in *G. lucidum* may speed up the process of understanding the lignocelluloses degradation mechanism for bioenergy applications.

## Results

### The genome characteristics of *G. lucidum*

The genome of monokaryotic *G. lucidum* was sequenced by whole genome shotgun strategy and produced 3,738 Mb clean data after filtering low quality and adapter contamination reads. The assembly was performed by SOAPdenovo genome assembler [22], firstly generated 1,724 contigs with N50 of 80,796 base pairs (bp) and then assembled into 634 scaffolds with N50 of 322,982 bp. The lengths of scaffolds ranged from 1,004 bp to 1,953,398 bp. Finally, we got a 39.9 Mb draft genome sequence for *G. lucidum*. Although we could not assemble these scaffolds into chromosomes, by using k-mer analysis, the expected genome size was 42.53 Mb, so these scaffolds covered 93.92% of the whole genome. The G+C content of the *G. lucidum* genome was 55.56%. The features of the assembled genome sequences are shown in Table 1.

**Table 1:** The characteristics of assembly scaffold and genome of *G. lucidum*.

**Scaffold characteristics**	
Total number	634
Total length (bp)	39,945,170
N50 (bp)	322,982
N90 (bp)	50,570
Max length (bp)	1,953,398
Min length (bp)	1,004
**Genome characteristics**	
Genome assembly (Mb)	39.9
Whole GC content (%)	55.56
Coding sequence GC content (%)	58.86
Number of protein-coding genes	12080
Coding sequence > = 100 amino acids	11522
Coding sequences/genome	43.31%
Average gene length (bp)	1959
Average coding sequence length (bp)	1435
Average exon length (nt)	230
Average intron length (nt)	100
Average number of exons per gene	6.25

### Repeat sequences in the genome

Five softwares were used to characterize transposons and the Tandem Repeat Finder was used to identify the tandem repeat sequences. Totally, we identified 2,025,242 bp repeat sequences, comprising 5.07% of the genome. No large scale dispersed segmental duplication was observed. Of them, tandem repeat sequences comprised 0.57% and transposable elements (TEs) were about 4.6% of the assembled genome. Among the TEs, long terminal repeats (LTR) and non-LTR transposons comprised 1.43% and 3.17% of the genome, respectively. Among the non-LTR transposons, DNA transposons (class II transposons) comprised 0.52% of the genome. The elements of DNA transposons mainly fell into four classes: Activator (hAT), Enhancer (En/spm), Harbinger and Mariner (Tc1).

### Predicted Gene models

By combining several different gene predictors (see methods), we identified 12,080 protein-coding gene models, 245 tRNA, 1 rRNA and 15 snRNA with a total length of 17,343,729 bp, accounting for 43.41% of the genome (Table 1). The gene density was 3.34 genes/10 kilobases (kb) and the average size of protein coding genes was 1,435 bp. Genes were typically with small exons (average 230 bp) and introns (average 100 bp), which were similar with other basidiomycetes [20]. There were average 6.25 exons in one gene. Notably, the G+C content in protein coding gene regions was 58.86%, slightly higher than the whole genome (55.56%) and other basidiomycetes [20].

Among the 245 tRNA genes, 10 tRNAs were pseudogenes and 141 tRNAs contained an intron. Forty six out of the 61 possible anti-codon tRNA were found, corresponding to the codons of 20 amino acids. The anti-codon usage and codon usage were shown in File S1. Except for several codons, the usage frequencies of most codons were proportional to the numbers of anti-codon proportion (File S2). For lacking of the other 18 anti-codons, we speculated anticodon repertoire in this genome was consistent with the normal wobble rules [23], which allow the following anticodon and codon pairings: I/ANN:NNU/NNC; GNN:NNU/NNC; UNN:NNA and CNN:NNG.

### Gene annotation

**Figure 1:**
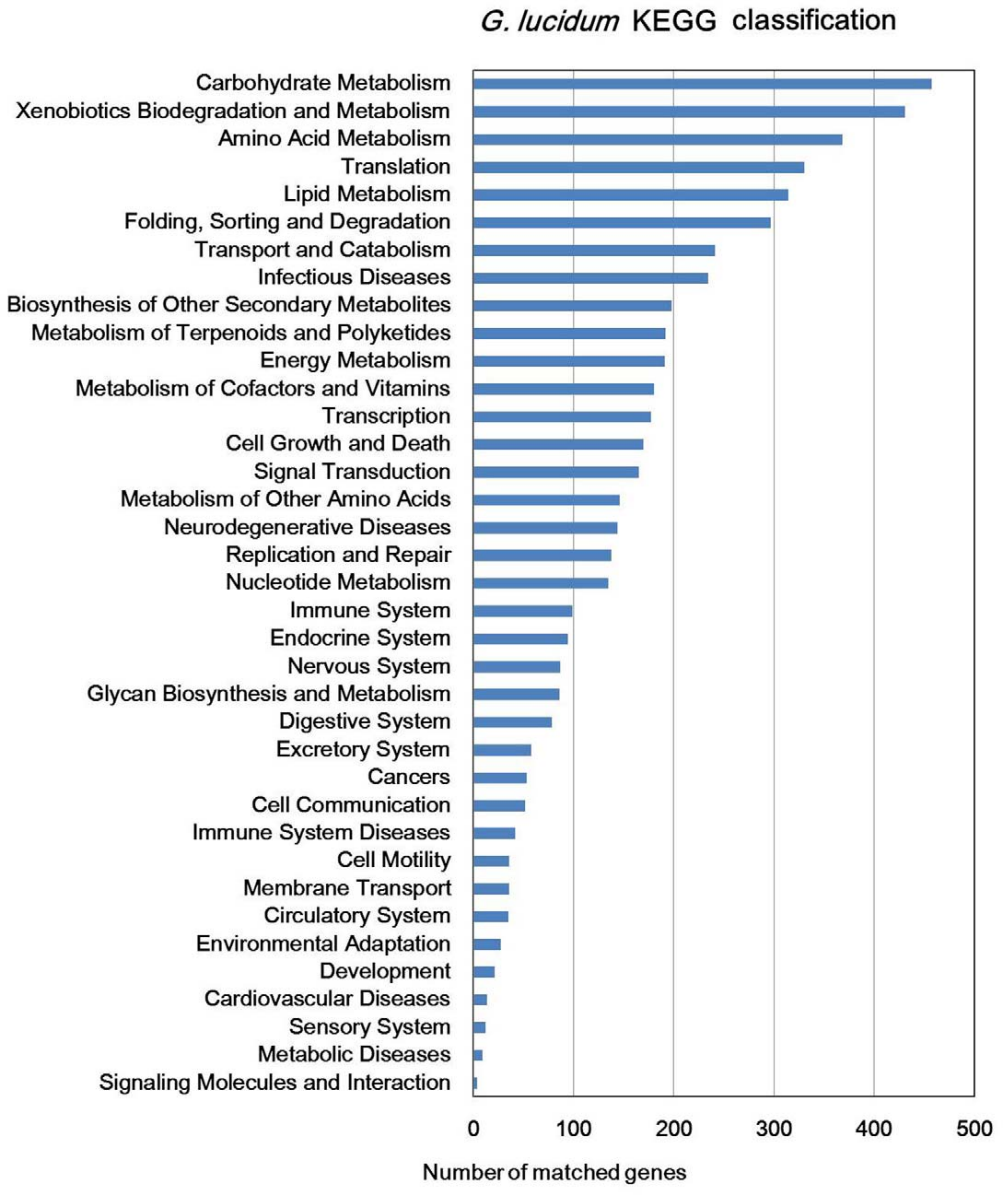
The KEGG function annotaion of *G. lucidum*. Distribution of Genes in different KEGG categories.

**Figure 2:**
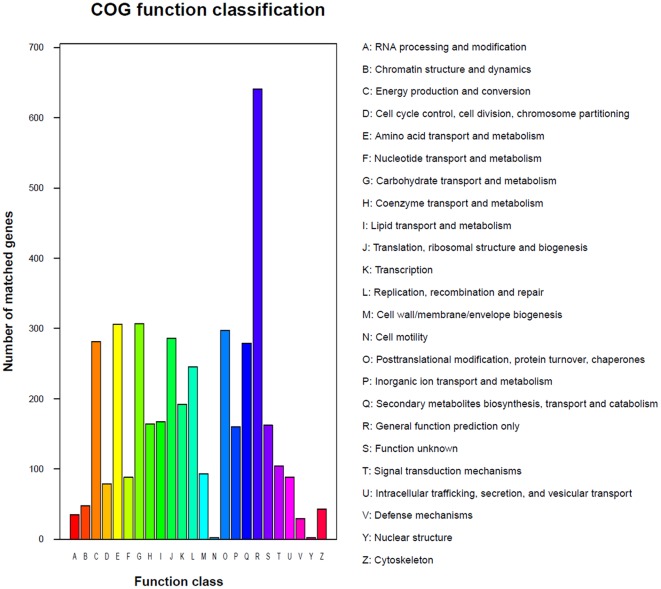
The COG function annotaion of *G. lucidum*. Distribution of Genes in different COG function classification.

By homology search, we mapped our predicted proteins to Gene Ontology (GO), 5,893 (49%) of which were assigned to GO terms, including 5,410, 1,738 and 4,034 genes mapped to the molecular function, cellular component and biological process categories, respectively. We also assigned 4,737 proteins to the Kyoto Encyclopedia of Genes and Genomes (KEGG) database. The annotations with KEGG, GO, InterPro, NCBI Clusters of Orthologous Groups of proteins (COG), NCBI non-redundant (nr), Pfam, SwissProt and TrEMBL protein databases were shown in File S1. KEGG function classification was shown in Figure 1, in which “Carbohydrate Metabolism”, “Xenobiotics Biodegradation and Metabolism” and “Amino Acid Metabolism” were the top 3 categories. Of these predicted genes in *G. lucidum*, up to 9,978, 6,436 and 9,981 showed a significant similarity (BLASTP, cut-off e-value<1e-7) to documented proteins in the NCBI nr database (Aug 2011), Swiss-Prot, and TrEMBL, respectively. As a result, about 83% of predicted proteins were similar to sequences in these public databases and only 2,094 genes were not similar to current public sequences, some of which might be *G. lucidum* specific genes. We further classed predicted genes into orthologous group (single-copy in *G. lucidum* and at least one other species ortholog), or paralogous group (multi-copy in *G. lucidum*). There were 4,689 orthologous genes and 5,510 paralogous genes by above definition. By the NCBI COG mapping, 3,509 (29%) proteins were assigned to COGs proteins (Figure 2). Similar to the KEGG annotation, some metabolisms and biosynthesis categories in COG were highly enriched.

Comparing with other published basidiomycetes, *G. lucidum* has many different biological characteristics, such as saprophytism, multiple triterpenoids and polysaccharides metabolites. To compare and find genes for *G. lucidum* specific characteristics, we performed comprehensive comparisons among *G. lucidum* and other published fungi genomes in the follow sections.

### Comparative genomics analysis of KEGG annotation

To make comprehensive comparison for KEGG annotations in fungi, KEGG pathway mapping was performed in 13 basidiomycetes and 5 ascomycetes (File S1). To facilitate comparison and show results in one table, we only showed the results of 8 basidiomycetes (7 in agaricomycotina and 1 in ustilaginomycotina) and 2 represented ascomycetes in the following analyses. In the second layer of KEGG pathway terms, we found fungi in agaricomycotina (including *G. lucidum*) had much more genes in each pathway than other fungi (Figure 1 and File S1). *G. lucidum* had relatively more genes in several pathways of metabolism and biosynthesis, such as “Metabolism of Terpenoids and Polyketides”, “Metabolism of Other Amino Acids” and “Xenobiotics Biodegradation and Metabolism”. In the third layer of KEGG, under the “Xenobiotics Biodegradation and Metabolism” pathway category, we found that *G. lucidum* and other Agaricomycotina fungi had relatively more proteins involved in several degradation pathways including pathways of aminobenzoate, bisphenol, dioxin and polycyclic aromatic hydrocarbon degradation (Table 2). There were about 190 genes involved in 3 of these degradation pathways in *G. lucidum* (Table 2). These results indicated that *G. lucidum* had strong ability of degradation. In addition, we also observed that “Metabolism of xenobiotics by cytochrome P450” and “Drug metabolism - cytochrome P450” sub-pathways had relative more genes in *G. lucidum*.

**Table 2:** The gene distribution of fungi in pathway “Xenobiotics Biodegradation and Metabolism”.

Pathway in KEGG	Pathway annotation	*Agaricomycotina*									
		*G. luc*	*F. pin*	*P. chr*	*S. com*	*P. ost*	*L. bic*	*C. cin*	*M. glo*	*P. tri*	*S. cer*
00362	Benzoate degradation	33	47	29	31	30	21	26	7	73	16
00627	Aminobenzoate degradation	190*	178	157	149	189	83	143	21	202	38
00364	Fluorobenzoate degradation	6	4	4	4	4	1	4	1	8	1
00625	Chloroalkane and chloroalkene degradation	71	85	78	108	67	43	39	14	79	27
00361	Chlorocyclohexane and chlorobenzene degradation	25	22	14	26	25	4	12	4	40	2
00623	Toluene degradation	13	14	8	12	15	4	12	4	28	1
00622	Xylene degradation	1	1	0	1	1	1	1	0	0	0
00633	Nitrotoluene degradation	0	0	1	0	1	3	1	1	1	0
00642	Ethylbenzene degradation	11	23	11	12	8	8	11	3	41	9
00643	Styrene degradation	14	11	14	8	9	5	7	3	23	4
00791	Atrazine degradation	3	3	3	1	2	1	1	0	7	1
00930	Caprolactam degradation	14	12	8	12	23	9	8	5	25	5
00351	1,1,1-Trichloro-2,2-bis(4-chlorophenyl)ethane (DDT) degradation	10	8	5	13	9	0	0	0	9	0
00363	Bisphenol degradation	196*	183	165	194	186	84	129	18	162	27
00621	Dioxin degradation	35*	21	27	24	12	9	12	0	19	0
00626	Naphthalene degradation	62	64	57	58	41	32	35	11	94	17
00624	Polycyclic aromatic hydrocarbon degradation	187*	148	151	136	150	59	99	11	132	8
00980	Metabolism of xenobiotics by cytochrome P450	44*	41	45	30	35	22	31	5	38	9
00982	Drug metabolism - cytochrome P450	38*	31	32	25	26	27	26	6	32	10
00983	Drug metabolism - other enzymes	14	14	12	14	16	15	20	6	17	12

* represents *G. lucidum* having relatively more genes than others. Abbreviations: *G. lui, Ganoderma lucidum; F. pin: Fomitopsis pinicola; P. chr: Phanerochaete chrysosporium; S. com: Schizophyllum commune; P. ost: Pleurotus ostreatus; L. bic: Laccaria bicolor; C. cin: Coprinopsis cinerea; M. glo: Malassezia globosa; P. tri: Pyrenophora teres; S. cer: Saccharomyces cerevisiae.*

In the fourth layer, *G. lucidum* had 27 KO terms with 1.5-fold genes more than other Agaricomycotina fungi (Table 3). Of them, the term K00490 (cytochrome P450) showed relatively more genes in *G. lucidum*. Since cytochrome P450 is a large group of enzymes involved in many important biosynthesis and metabolism pathways, we further identified and performed comparison about P450 genes at genome level in these fungi. We found that the numbers of P450 genes in agaricomycotina were much more than those in other subphylums of basidiomycota and in ascomycota (Table 4). G. *lucidum* had 222 putative P450 genes, which was the largest one in the 10 represented fungi and the top 3 in all the 18 fungi we analyzed.

**Table 3:** KO families showing relatively more genes in *G. lucidum* genome as compared to other Basidiomycota fungi.

Pathway in KEGG	KO description	*G. luc*	*F. pin*	*P. chr*	*S. com*	*P. ost*	*L. bic*	*C. cin*
K00490	CYP4F; cytochrome P450, family 4, subfamily F	48	41	12	20	14	20	16
K00480	E1.14.13.1; salicylate hydroxylase	34	20	27	23	11	8	11
K01046	E3.1.1.3; triacylglycerol lipase	26	21	14	10	19	14	13
K01279	TPP1, CLN2; tripeptidyl-peptidase I	24	31	10	5	7	7	2
K04125	E1.14.11.13; gibberellin 2-oxidase	22	17	4	4	10	1	1
K10866	RAD50; DNA repair protein RAD50	22	14	21	12	7	6	8
K01183	E3.2.1.14; chitinase	21	15	10	13	12	11	9
K01423	E3.4.-.-;	19	8	17	26	12	5	12
K00140	malonate-semialdehyde dehydrogenase/methylmalonate-semialdehyde dehydrogenase	18	1	45	1	1	2	1
K01528	DNM; dynamin GTPase	11	4	3	4	4	3	3
K00218	E1.3.1.33; protochlorophyllide reductase	8	6	4	2	2	3	5
K01190	lacZ; beta-galactosidase	8	2	3	4	5	0	0
K03942	NDUFV1; NADH dehydrogenase (ubiquinone) flavoprotein 1	7	2	1	2	2	2	2
K06148	ABCC-BAC; ATP-binding cassette, subfamily C, bacterial	7	2	5	3	2	4	6
K01044	E3.1.1.1; carboxylesterase	6	0	2	4	4	3	5
K02831	RAD53; ser/thr/tyr protein kinase RAD53	6	1	1	0	2	2	1
K00119	E1.1.99.-;	5	0	2	1	2	0	3
K00129	E1.2.1.5; aldehyde dehydrogenase (NAD(P)+)	5	2	2	2	2	0	1
K01082	E3.1.3.7; 3′(2′), 5′-bisphosphate nucleotidase	5	3	1	4	1	1	1
K09202	regulatory protein SWI5	5	2	1	1	3	2	1
K09553	STIP1; stress-induced-phosphoprotein 1	5	0	1	1	0	1	5
K00135	E1.2.1.16; succinate-semialdehyde dehydrogenase (NADP+)	4	1	1	1	1	1	1
K01539	ATP1A; sodium/potassium-transporting ATPase subunit alpha	4	4	1	0	1	2	0
K02133	ATPeF1B, ATP5B; F-type H+-transporting ATPase subunit beta	4	1	1	1	1	1	1
K10590	TRIP12; E3 ubiquitin-protein ligase TRIP12	4	1	1	1	1	1	1
K12388	SORT1; sortilin	4	1	1	1	1	2	1
K09753	CCR; cinnamoyl-CoA reductase	3	0	1	0	0	0	2

The abbreviations of species were the same with Table 2. The number of genes in *G. lucidum* of each KO of is 1.5 fold more than the average of the other **Basidiomycota** fungi.

**Table 4:** The gene distribution of fungi in P450 family and GST family.

		*G. luc*	*F. pin*	*P. chr*	*S. com*	*P. ost*	*L. bic*	*C. cin*	*M. glo*	*P. tri*	*S. cer*
P450		222*	196	154	120	160	113	143	12	97	6
GST											
	EFBy	1	1	1	1	1	2	1	2	1	3
	GTE	4	10	5	9	7	3	14	0	2	0
	GTT1	1	1	0	2	1	0	2	0	1	1
	GTT2	8	4	3	6	3	11	6	0	0	1
	MAK16	1	1	1	1	1	0	1	1	1	1
	omega	18*	7	8	8	8	3	5	1	4	3
	URE2p	6	8	9	0	1	1	2	1	2	1
	TOTAL	39*	32	27	27	22	20	31	5	11	10

* represents *G. lucidum* having the most genes than others. The abbreviations of species were the same with Table 2.

Under the “Metabolism of xenobiotics by cytochrome P450” pathway, we found the glutathione S-transferases (GST, EC 2.5.1.18), a kind of well-known detoxification enzymes [24], were greatly enriched in *G. lucidum* compared with other fungi. According to the classification of Morel *et al.* [25], we investigated the GSTs distribution in six known classes (GTT1, GTT2, URE2p, Omega, EFBγ, MAK16) and a new class (GTE) of all fungi in this study. Under the relatively strict cutoff (BLASTP e-value<1e-10 and identity>30%), we found 39 GST genes in *G. lucidum*, which was the highest GST gene numbers among all fungi we analysed. Notably, *G. lucidum* had 18 genes in the Omega subfamily, which were much more than other fungi (Table 4).

### The pathway of triterpenes synthesis

The triterpenes have been reported of great importance in *G. lucidum* because of their significant roles in immune regulation and other biological activities [4]–[7]. In plants, there are two pathways to synthesize terpenoids: the Mevalonate (MVA) pathway and methylerythritol 4-phosphate/deoxyxylulose 5- phosphate (MEP/DOXP) pathway. It has been suggested that the MEP/DOXP pathway do not exist in fungi [8]. We checked the *G. lucidum* genes in the “terpenoid backbone biosynthesis (map00900)” pathway and found that the genes only distributed in MVA pathway, no gene existed on the MEP/DOXP pathway (File S2). The similar results were found in other basidiomycetes and ascomycetes. These observations verified that terpenoid backbone biosynthesis only could be through the MVA pathway in fungi at the genome level.

**Figure 3:**
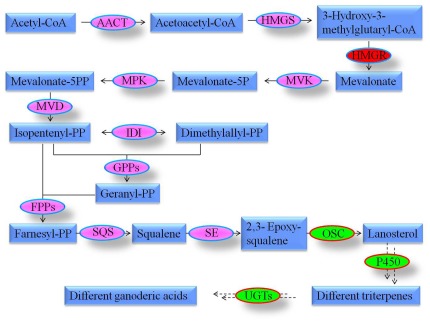
Putative ganoderic acid biosynthesis pathway in *G. lucidum*. Enzymes involved in this pathway are: AACT: acetyl-CoA acetyltransferase, [EC:2.3.1.9], K00626; HMGS: 3-hydroxy-3-methylglutaryl-CoA synthase, [EC:2.3.3.10], K01641; HMGR: 3-hydroxy-3-methylglutaryl-CoA reductase, [EC:1.1.1.34], K00021; MVK: mevalonate kinase, [EC:2.7.1.36], K00869; MPK: phosphomevalonate kinase, [EC:2.7.4.2], K00938; MVD: pyrophosphomevalonate decarboxylase, [EC:4.1.1.33], K01597; IDI: isopentenyl-diphosphate isomerase, [EC: 5.3.3.2], K01823; GPPs: geranyl diphosphate synthase, [EC: 2.5.1.1], K00787, K00804; FPPs: farnesyl diphosphate synthase, [EC: 2.5.1.10], K00787, K00804; SQS: squalene synthase, [EC: 2.5.1.21], K00801; SE: squalene monooxygenase, [EC: 1.14.99.7], K00511; OSC: 2, 3-oxidosqualene-lanosterol cyclase, [EC: 5.4.99.7], K01852; P450: cytochrome P450, [EC: 1.14.-.-]; UGTs: uridin diphosphate glycosyltransferases, [EC: 2.4.1.-]. Ingredients are in blue box. Limited enzymes are in red oval, key enzymes are in green oval while the other enzymes are in pink oval. Solid arrows and broken arrows represent single and putative multiple enzymatic steps respectively.

By integrating MVA pathway in KEGG and plant triterpenoid saponins biosynthesis from literatures, we summarized the potential triterpenoids biosynthesis pathway in *G. lucidum* (Figure 3). The pathway contained 14 steps catalyzed by different enzymes. The first 11 steps are the common steps for terpenoid skeleton biosynthesis and the last 3 steps may be specific for different triterpenes in different species. We identified and summarized the enzymes in the ganoderic acids (GAs) biosynthesis in table 5 from the *G. lucidum* genome, which includes 6 putative UDP-glycosyltransferases (UGTs) genes (Table 5).

**Table 5:** The putative genes involved in triterpene biosynthesis.

Gene full name	Abbr.	Enzyme	KO	Putative gene
acetyl-CoA acetyltransferase	AACT	EC:2.3.1.9	K00626	G_lucidum_10003032
3-hydroxy-3-methylglutaryl-CoA synthase	HMGS	EC:2.3.3.10	K01641	G_lucidum_10008701
3-hydroxy-3-methylglutaryl-CoA reductase	HMGR	EC:1.1.1.34	K00021	G_lucidum_10003589
mevalonate kinase	MVK	EC:2.7.1.36	K00869	G_lucidum_10009892
phosphomevalonate kinase	MPK	EC:2.7.4.2	K00938	G_lucidum_10010135
pyrophosphomevalonate decarboxylase	MVD	EC:4.1.1.33	K01597	G_lucidum_10005090
isopentenyl-diphosphate isomerase	IDI	EC:5.3.3.2	K01823	G_lucidum_10001705
geranyl diphosphate synthase	GPPs	EC: 2.5.1.1	K00787/K00804	G_lucidum_10002724;
				G_lucidum_ 10008471;
				G_lucidum_10004225
farnesyl diphosphate synthase	FPPs	EC: 2.5.1.10	K00787/K00804	G_lucidum_10002724;
				G_lucidum_ 10008471;
				G_lucidum_10004225
squalene synthase	SQS	EC 2.5.1.21	K00801	G_lucidum_10005172
squalene monooxygenase	SE	EC 1.14.99.7	K00511	G_lucidum_10007072
2, 3-oxidosqualene- lanosterol cyclase	OSC	EC 5.4.99.7	K01852	G_lucidum_10008645;
				G_lucidum_10008646
cytochrome P450	P450	EC: 1.14.-.-		222 putative genes
UDP-glucosyl transferase	UGT	EC: 2.4.1.-		G_lucidum_10003239;
				G_lucidum_10003516;
				G_lucidum_10009503;
				G_lucidum_10009504;
				G_lucidum_10010093;
				G_lucidum_10010094

**Figure 4:**
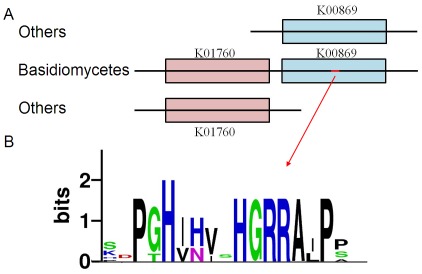
The features of metC-MVK in basidiomycetes. (A) The metC-MVK matched two enzymes at the same time and some of them have an addition sequence in the middle of K00869 (red line). (B) The added conservative sequence in 7 of 13 Basidiomycota fungi.

Interestingly, we observed a fusion gene in the triterpenes biosynthesis pathway in 12 Basidiomycete fungi except for *L. bicolor*. The N-terminal of the protein was similar to the enzyme K01760 (KEGG ID, cystathionine beta-lyase, metC), while the C-terminal was similar to another enzyme K00869 (KEGG ID, mevalonate kinase, MVK) which is an enzyme in the triterpenes biosynthesis pathway (Figure 4A). These proteins were referred as metC-MVKs in the following. In all other species except basidiomycetes, no such a protein matched the two enzymes at the same time. By multiple sequence alignment of the fusion protein in basidiomycetes, the average length of metC-MVKs was ∼886 aa and about half of it matched with K01760 and half matched with K00869. The metC-MVK protein was the only homologous protein with K00869 in our analyzed basidiomycetes, so they should be involved in terpenoid backbone biosynthesis functioning as K00869 in other species. We also noticed that this metC-MVK gene was the only gene which best hit K01760. In addition, seven of the 12 Basidiomycota fungi had a 16 amino acids conserved insertion sequence in the middle of the MVK regions of the metC-MVK gene (Figure 4B).

### Phylogeny of *G. lucidum* and multigene families

**Figure 5:**
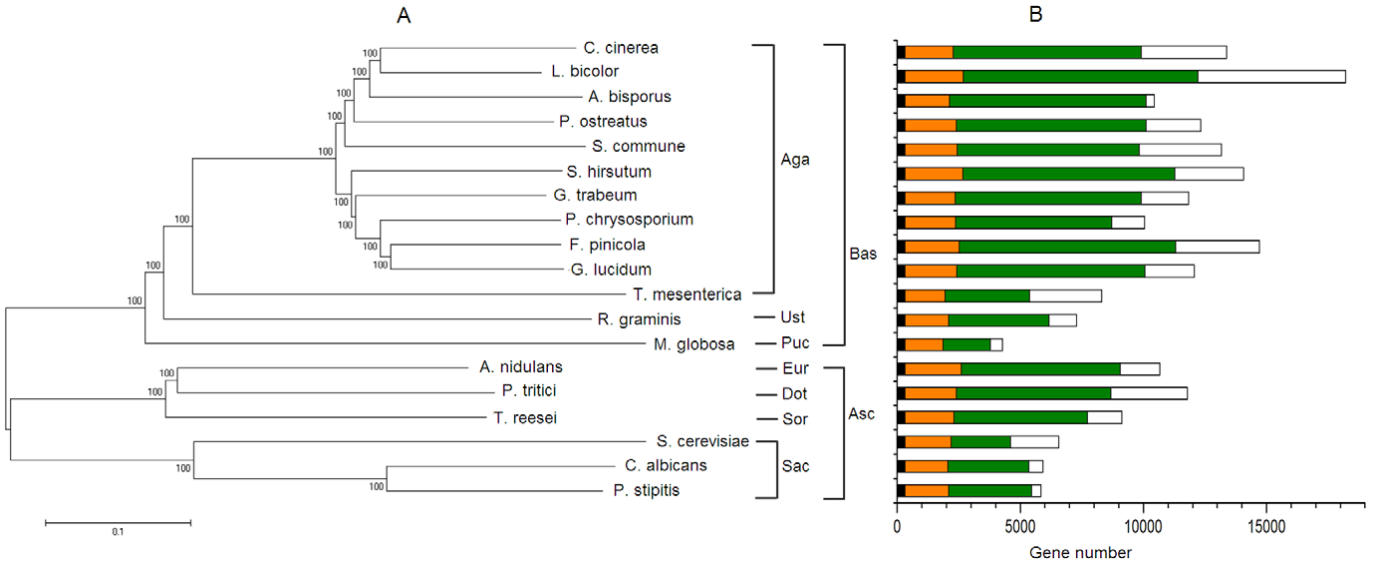
Phylogeny tree of 19 fungi and genes in their genomes. (A) The Neighbor Joining tree (NJ) was constructed with 1,000 bootstrap replications from a concatenated alignment of 323 single-copy proteins. (B) Bars represent a comparison of the gene content of all these species in the corresponding position in NJ tree. Bars are subdivided to indicate different types of homology relationships. Black: genes that are found only one copy in all these fungi (323 genes); Orange: genes that were in all species but maybe more copies in some species; Green: genes presenting in more than one fungus but not in all these fungi; White: species-specific genes with no detectable homologs in other species genes. Abbreviations: Aga, Agaricomycotina; Ust, Ustilaginomycotina; Puc, Pucciniomycotina; Eur, Eurotiomycetes; Dot, Dothideomycetes; Sor, Sordariomycetes; Sac, Saccharomycotina; Bas, Basidiomycota; Asc, Ascomycota.

The phylogenetic tree constructed by concatenated sequences alignments showed that *G. lucidum* was close to another polyporale fungus *Fomitopsis pinicola* in the evolutionary relationship among all our analyzed fungi (Figure 5A). In the all-to-all BLASTP analysis, 9,278 predicted proteins of *G. lucidum* showed high sequence similarity with that of *F. pinicola* (BLASTP, cut-off e-value<1e-7). Following, 9,013 and 8,872 predicted proteins showed significant sequence similarity to that of *Gloeophyllum trabeum* and *Stereum hirsutum*, which were all in polyporales.

In order to investigate the gene family expansion in *G. lucidum*, we performed analyses for multi-gene families, which were generated from proteins in 8 Agaricomycotina species. In total, 10,720 gene families (File S1) containing at least two members were generated using the Tribe-MCL tool, of which 5,947 families had at least one *G. lucidum* gene and 1,487 families had at least two *G. lucidum* genes. The largest gene family had 517 genes and 126 of them were *G. lucidum* genes. In 3,540 lineage specific gene families, 287 families were *G. lucidum* specific (File S1). The number was very similar to that of *G. trabeum* and much lower than other basidiomycetes. *L. bicolor* had the largest (947) lineage-specific gene families, which may be related to its biggest genome size among our analyzed basidiomycetes. The distributions of genes with different copies or species-specific are shown in Figure 5B.

Besides the lineage specific gene families, the evolutionary changes in the size of each gene family were performed using CAFE program. As a result, we found that among the 7,180 non-lineage specific gene families for *G. lucidum*, 636 of them were expanded and 994 of them had undergone contraction. The function of the most abundant gene family was uncharacterized for lack of available annotation, while genes in the second most abundant gene family encoding proteins with a P450 domain (File S1). The expanded and contracted gene families and their annotations were shown in File S1.

### *G. lucidum* has multiple copy *het*-like genes

Among the 287 *G. lucidum* specific gene families (File S1), the largest *G. lucidum* specific gene family had 101 genes and 89 of them had the *HET* (heterokaryon incompatibility protein) domain, which is related to vegetative (or heterokaryon) incompatibility (VI). It is surprised that so many *het*-like genes were found in *G. lucidum*, while few *het* genes were reported in other fungi. In PFAM database, there are three vegetative incompatibility related domains, which are *HET*, *Het-c* and *HET-s*. Since the *HET* related studies were mostly reported in fungi *P. anserina* and *N. crassa*, we added them in our analyzed fungi list to identify the *HET* genes. Thus, in total, we scanned 7 ascomycetes and 13 basidiomycetes for genes with the three *het* related domains. The results were shown in File S1 and only *P. anserina* had one *HET-s* domain. The number of *Het-c* genes in each species was always 0–2 and the highest one was four. While the number of genes with *HET* domain varied from 0 to 126. It seems that *het-c* and *HET-s* are comparatively conserved. *G. lucidum* had two genes with *Het-c* domain and 96 genes with *HET* domain, which was much more than other basidiomycetes and most ascomycetes. In the comparison, we also observed that there were 62 and 126 *HET*-like genes in *N. crassa* and *P. anserine* in which the number of *het* genes were reported for 11 and 9, respectively [26]. Thus, some of the *het*-like genes may play roles in other function not for VI, such as *mat a/A* for mating in *N. crassa* and *het c* for ascospore formation in *P. anserina* [26], [27]. Therefore, it may be a complex system not only one locus affect the VI. Since one of *het-c* loci in *P. anserina* is similar to the glycolipid transfer protein (GLTP) [27], the GLTP domain was also scanned in this study. We found two genes G_lucidum_10005152 and G_lucidum_10009654 with a GLTP domain, which also might be *het-c* genes.

These *HET* genes in *G. lucidum* encoded proteins with an average length of 2,686 amino acids and did not uniformly spread across the genome. The 98 genes were located on 45 scaffolds (total 634 scaffolds). Of them, 13 scaffolds had more than two *HET* genes and three scaffolds had more than 10 *HET* genes, suggesting the expansion of *HET* genes might have undergone tandem duplications. Except for the *HET* domain, some *HET* genes also had other domains, such as, adh_short,Aldo_ket_red, ICMT,Nup96, p450, SUR7, and WD40.

### Function annotation of putative CAZymes

CAZy is a carbohydrate-active enzymes (CAZymes) database (http://www.cazy.org/) [28], which classifies the CAZymes into 5 major modules: Glycoside Hydrolases (GH), Glycosyl Transferases (GT), Polysaccharide Lyases (PL), Carbohydrate Esterases (CE), and Carbohydrate-Binding Modules (CBM). We mapped our analyzed fungi genomes to CAZy to study the members and features of these Carbohydrate-active enzymes. The results revealed that the gene numbers in the 5 major modules of CAZymes were similar in Agaricomycotina fungi, while much fewer in Ustilaginomycotina and Ascomycota fungi. *G. lucidum* possessed a wide spectrum of CAZymes responsible for the biosynthesis, degradation and modification of oligo- and polysaccharides, and of glycoconjugates (Table 6). The GHs and CEs in *G. lucidum* showed a little more than average count, while GTs, CBMs and PLs showed less than the Agaricomycotina average (Table 6).

**Table 6:** The gene distribution of different fungi in CAZymes family.

	CAZY	GH	GT	CBM	CE	PL	Total
**Agaricomycotina**	*G. lucidum*	216*	56	34	40*	3	349
	*F. pinicola*	169	61	13	33	1	277
	*P. chrysosporium*	149	54	42	23	1	269
	*S. commune*	204	65	38	42	13	362
	*P. ostreatus*	221	57	64	47	13	402
	*L. bicolor*	105	59	12	27	1	204
	*C. cinerea*	189	65	56	49	9	368
	Above average	179	60	37	37	6	319
**Ustilaginomycotina**	*M. globosa*	13	34	1	6	1	55
**Dothideomycetes**	*P. tritici*	221	91	43	60	10	425
**Saccharomycotina**	*S. cerevisiae*	46	68	14	4	0	132

* represents *G. lucidum* having relatively more genes than the average of 7 Agaricomycotina fungi. Abbreviations: GH, Glycoside Hydrolases; GT, GlycosylTransferases; CBM, Carbohydrate-Binding Modules; CE, Carbohydrate Esterases; PL, Polysaccharide Lyases.

### Function annotation of putative FOLymes

To assess the degradation in genomic level, proteins of *G. lucidum* were aligned to proteins in the FOLy (Fungal Oxidative Lignin enzymes) database, which collects and classifies enzymes involved in lignin catabolism. The FOLymes mainly comprise two families, lignin oxidases (LO families) and lignin-degrading auxiliary enzymes (LDA families) that generate H_2_O_2_ for peroxidases. *G. lucidum* contained a total of 48 members in FOLymes (24 genes in LO families and 24 genes in LDA families, Table 7) which was more than brown-rot fungi *F. pinicola,G. trabeum* and the fungi without ligninolytic activity, such as *Malassezia globosa, Pyrenophora teres* and *Saccharomyces cerevisiae*. In contrast, it had fewer FOLymes than the coprophilic fungus *Coprinopsis cinerea* (59 FOLymes) and the white-rot fungus *Pleurotus ostreatus* (72 FOLymes). While *G. lucidum* had the largest number of lignin oxidases (LO families). The LO families can further divided into 3 subfamilies, which are laccases (LO1), lignin peroxidases, manganese peroxidases, versatile peroxidases (LO2) and cellobiose dehydrogenases (CDHs; LO3). *G. lucidum* contained 16 laccase genes (LO1), 7 peroxidase (LO2) and 1 cellobiose dehydrogenase (LO3). For the seven peroxidases (LO2) genes in *G. lucidum*, two of them located at scaffold 10 and 3 located at scaffold 79 which maybe form a gene cluster. While LDA families have 7 sub families, *G. lucidum* contained 10 aryl-alcohol oxidase (LDA1), 9 copper radical oxidase (LDA3), 3 glucose oxidase (LDA6) and 2 benzoquinone reductase (LDA7). Similar to most other fungi, no LDA2, LDA4 or LDA5 gene was found in *G. lucidum*. The major fungi contained multi-copy genes in LO1 except *P. chrysosporium*, which had only 1 LO1 gene but 16 LO2 genes.

**Table 7:** The gene distribution of FOLymes in *G. lucidum* and other fungi.

FOLYmes	*G. luc*	*F. pin*	*P. chr*	*S. com*	*P. ost*	*L. bic*	*C. cin*	*M. glo*	*P. tri*	*S. cer*
LDA1	10	7	4	3	29	4	27	1	8	0
LDA2	0	0	0	0	0	0	0	0	1	0
LDA3	9	4	7	2	16	5	6	1	2	0
LDA4	0	0	1	1	0	0	0	0	0	0
LDA6	3	1	3	11	4	2	4	3	4	0
LDA7	2	1	4	4	2	2	3	0	1	3
LO1	16	6	1	5	12	11	17	2	7	2
LO2	7	1	16	0	8	1	1	0	0	0
LO3	1	0	1	1	1	0	1	0	2	0
total	48	20	37	27	72	25	59	7	25	5

The abbreviations of species were the same with Table 2. LO1, laccases; LO2, peroxidases; LO3, cellobiose dehydrogenases; LDA1, aryl alcohol oxidases; LDA2, vanillyl-alcohol oxidases; LDA3, glyoxal oxidases; LDA4, pyranose oxidases; LDA5, galactose oxidases; LDA6, glucose oxidases; LDA7, benzoquinone reductases.

## Discussion

*G. lucidum* is one of the most famous traditional medicines in China and it is also an important fungus in cellulose and lignin degradation with potential ability in energy production. The genome sequencing and annotation of *G. lucidum* are crucial for its function and comparative genomics research. Here we selected the most commonly used *G. lucidum* in China and sequenced its genome sequence by Solexa technology. We assembled the sequences into 634 scaffolds in 39.9 Mb sequences represented about 93.92% of the whole genome and annotated 12,080 gene models at genome level. We noticed that JGI (DOE Joint Genome Institute) also have sequenced and annotated the genome of a North American isolate *G. lucidum*. The predicted gene models of our sequenced genome were very similar with JGI genome annotation, which indicated the quality of our sequence and annotation were reliable.

The *G. lucidum* genome was characteristics of a relatively high GC content and less TEs compared with other sequenced fungi. In *G. lucidum* genome, we only identified 4.6% TE sequences of the genome, which was much less than other reported fungi, for example, *Laccaria bicolor* (21%) [20] and *Tuber melanosporum* (58%) [29]. To check whether the lower repeat percentage was caused by methodology or not, we had used our approaches to identify the repeat sequences in *L. bicolor* and found a similar percentage of repeat sequences with reported [20]. This confirmed that the TEs in *G. lucidum* were really much less than other fungi. Although the repeat sequence percentage was different, the genome size, gene length, and gene annotation of *G. lucidum* were similar by comparing with other Basidiomycota fungi.

### Ganoderic acids biosynthesis pathway

Ganoderic acid (GA), a kind of triterpenoids, is the main medicinal component of *G. lucidum* with function of anti-tumor, immuno-reglulation, and anti-oxidant *et al.* [8]. Currently, studies on triterpenoid biosynthesis are mostly performed in plants; the detailed biosynthesis pathways in fungi are still unclear. Shiao [30] proved that GAs were synthesized via MVA pathway by using isotopic tracer experiments. In *G. lucidum* genome, we found genes only involved in MVA pathway but not MEP pathway, which is another terpenoids biosynthesis pathway in plants. Thus, at the genome level, we confirmed that the terpenoids biosynthesis of *G. lucidum* was only via MVA pathway, not MEP pathway. Interestingly, we identified a fusion gene (metC-MVK) in basidiomycetes, half of which was homologous to MVK enzyme in terpenoids biosynthesis and half of which was similar to metC enzyme, a cystathionine beta-lyase. In animal and even Ascomycota fungi, they were two separate genes, implying the appearance of metC-MVK occurred in the ancestor of Basidiomycota fungi. The study of the reason and function of the gene fusion is going on.

We have characterized all the enzymes in the terpenoid backbone biosynthesis in *G. lucidum* (Table 5). In triterpenoid synthesis, 2, 3-epoxysqualene is the precursor and the difference of cyclization, oxidation, hydroxylation and glycosylation leads to different triterpenoids [31]. However, there is little knowledge about the pathway that lies in downstream of cyclization. It has been reported that these modification are carried out by cytochrome P450, glycosyltransferases and other enzymes [31]. P450 are speculated to be involved in a wide range of modification, including oxidation and hydroxylation in the synthesis of triterpenoids [32]. Due to the large number and diversity, it is difficult to identify their specific functions based on homology. There were only four P450 genes reported to involve in the biosynthesis of triterpenoid saponins [32]–[34]. So far no P450 genes have been cloned in GAs biosynthesis of *G. lucidum*, we characterized 222 genes encoding P450 enzymes and 21 of them were very similar with the four known P450 genes. The plentiful of putative P450 genes provided the potential of different oxidation and hydroxylation, thus formed plentiful GAs in *G. lucidum*.

Glycosylation, which transfers the active saccharides to the triterpenoid backbones and alters its physiological activity [35], is the last and key modification in GAs biosynthesis. UGTs are reported to contribute the glycosylation in triterpenoid biosynthesis and so far only six UGTs are experimentally identified in the triterpenoid biosynthesis [33], [34]. By searching against known UGTs sequences [32], we found six putative UGTs in *G. lucidum* genome, which might be responsible for the glycosylation modification in the GAs biosynthesis in *G. lucidum*. Among these six putative UGTs, the gene (G_lucidum_10009504) was highly similar with UGT73K1, which glycosylated both triterpenoids and (iso)flavones in *Medicago truncatula* [36]. Extended searching other fungi against known UGTs sequences, we found that there were several UGT homologs in the Agaricomycotina fungi but none of them in the Ascomycota fungi. This might suggest that other glycosylation enzymes instead of UGTs were applied in the triterpenoid synthesis of ascomycetes.

### Genes related to biodegradation in *G. lucidum*

Besides its medicinal and economic value, limited studies were performed regarding the function of biodegradation of *G. lucidum* so far. JGI sequenced its genome due to its ability in wood degradation and potential value in bioenergy production. In our analysis, we found that many genes might be involved in bio-degradation in *G. lucidum* genome. *G. lucidum* could degrade the major components of plant cell walls including cellulose, hemicelluloses and lignin. After predicted the CAZymes in *G. lucidum*, 216 putative GH genes and 56 putative GT genes were found. The number of GHs was comparatively larger than GTs. This may be related to its lifestyle, in which its survival depends on decomposed lignocelluloses, thus decomposing polysaccharides is more important than constructed. Similar phenomenon was observed in *P. chrysosporium* [29].

Lignin is the second most abundant renewable organic polymer and its degradation has great potential value to reproductive energy. Thus the research about lignin degradation especially in white rot fungi is increasing. We checked the FOLymes enzymes in *G. lucidum* and found 16 laccase (LO1), 7 peroxidases (LO2) and 9 glyoxal oxidases (LDA3) genes. Laccase is one of the most applied ligninolytic enzymes, for its broad substrate specificity and the generation of water as by-product [37]. Except *P. chrysosporium* had single copy of LO1 genes, all of other fungi contained more than one LO1 genes suggesting LO1 played an important role in lignin degradation of fungi. Besides its essential for wood and lignin decomposition, LO1 is also involved in other functions, such as pigments synthesis, fruiting bodies and spores formation [38]. Makela *et al.* reported that multiple *lip*, *mnp* and *lac* transcripts were coexpressed in *P. radiate*, indicating the potential synergy of the fungal LO1 and LO2 upon white rot fungal decay of wood [39].

LO2 mainly existed in white rot and wood-colonising basidiomycetes (*P. chrysosporium*, *P. ostreatus*, and *G. lucidum*). It has been reported that glyoxal oxidases (LDA3) were inactive unless they coupled to LO2 reaction [40]. Here, we identified 9 LDA3 in *G. lucidum*. Considering the total number of LO2 and LDA3 genes in all fungi we analyzed, *P. ostreatus* (24 genes) and *P. chrysosporium* (23 genes) were the top two fungi and were reported with strong ligninolytic ability. *G. lucidum* had the third largest number of LO2 and LDA3 genes (16 genes), which may suggest its strong ligninolytic ability. In addition, in *G. lucidum* genome we also found 10 LDA1 gene, which could reduce the level of radical compounds and quinonoids produced by LO1, leading an oxidative enzyme system with LO1 [41].

Moreover, *G. lucidum* showed rich P450 family, abundant GST enzymes (mainly Omega class) and abundant genes involved in “Xenobiotics Biodegradation and Metabolism” pathways. Some of these pathways are related to the degradation of refractory compounds, such as dioxin and naphthalene. These enzymes have potential to degrade various industrial pollutants [42]. In view of the extensive contamination of the environment by persistent and toxic chemical pollutants, the utility of the degradation of fungi including *G. lucidum* may be an attractive and effective approach on pollution controlling.

## Materials and Methods

### Strains and culture conditions

A fruiting body of *G. lucidum* was collected from oak at Hengshan, Hunan province, China, on May 21th, 2001. No specific permits were required for the described field studies. We confirmed that the location was not privately-owned or protected in any way. The *G. lucidum* was deposited at the edible fungi institute of Hunan agricultural university (Changsha, Hunan, China). Basidiospores from the fruiting body of *G. lucidum* were collected by hood. Single spores were separated by micro-manipulation of basidiospores and allowed to geminate on PDA (Potato Dextrose Agar) enrichment medium (20% potato, 2% dextrose, 0.2% yeast extract, 0.2% peptone,0.3% monopotassium phosphate, 0.15% magnesium sulfate and 2% agar) at 25°C, dark. Germination started 4–8 days after plating. After 3–5 days growth, clamp connection was observed by using optical microscopy. Forty-six single basidiospores with no clamp connection were isolated and transferred individually to fresh dishes, sealed and stored at 4°C after 10–15 days growth. For genomic DNA isolation, single basidiospore named P9 which grew well was cultured in potato dextrose agar enrichment medium without agar at 25°C, dark, shaken at 120 r/min, for 8–10 days.

### DNA isolation, genome sequencing and assembly

Genomic DNA of *G. lucidum* was isolated by improved cetyl trimethylammonium bromide (CTAB) method [43] and sequenced using a whole-genome shotgun strategy. All data were generated by paired-end sequencing of cloned inserts with two different insert sizes (200 bp, 6000 bp) using Illumina Hiseq2000 Sequencer at BGI-Shenzhen. After removing the low complexity, low quality, adapter and duplication contamination raw reads, the clean reads were assembled using the whole-genome *do novo* assembler SOAPdenovo [22].

### Annotation methods

Protein coding gene models were predicted using *de novo* prediction tools Genscan [44],Augustus [45] and GeneMark-ES [46] and homology based gene prediction tool Genewise [47] with the default parameters. The homology-based and *de novo* gene sets were merged to form a comprehensive and non-redundant reference gene set by Glean [48].

The functionally annotation of predicted gene models were mainly based on homology to known annotated genes and BLAST was the mainly used tool in our analyses. We aligned all protein models by BLASTP to SwissProt, TrEMBL, and NCBI nr, InterPro, Pfam [49] and also mapped them onto functional terms, including GO [50], COGs [51] and KEGG pathways [52] (BLASTP cut-off e value<1e-7). Since each gene mapped to different database sequences, there may be multiple aligned results meeting the cut-off, the annotations of the sequences with the best score were chosen to be the annotation of the gene in *G. lucidum*.

Transposons were identified by aligning the assembled results with the known sequences of the transposon library. The specific method was made through the RepeatMasker software (http://www.repeatmasker.org, using Repbase database [53]), RepeatProteinMasker software (using the transposon protein library that comes with RepeatMasker) and 3 other tools LTR-FINDER [54], RepeatScout [55] and PILER [56] with default parameters. Tandem Repeat Finder [57] software was used to predict tandem repeats.

rRNAs were identified by BLAST against the rRNA libraries or predicted by using rRNAmmer [58] software. tRNAscan-SE [59] software was used to detect tRNA regions and its secondary structures. Other non-coding RNAs such as miRNA, sRNA and snRNA were predicted by Rfam.

### KEGG pathway analysis

To compare the pathway annotation of *G. lucidum* with other fungi, we also mapped genes in other fungal genomes to KEGG database. The Basidiomycota comprises three taxa (agaricomycotina, ustilaginomycotina and pucciniomycotina). The Ascomycota includes four taxa– eurotiomycetes, dothideomycetes, sordariomycetes and saccharomycotina. For comparative genome analysis, we selected 12 basidiomycetes, 5 ascomycota and downloaded their genomic gene models [20], [29], [60] (File S1). Then, we compared the number of genes in each KEGG terms among all these genomes. Terms in KEGG are divided into four layers. The first layer consists of seven sections, including “Metabolism”, “Genetic information processing”, “Environmental information processing” and so on. Each section is further divided into several small entries, which are the second layers. The third layer is the specific pathway map and the fourth layer includes specific genes in each pathway. We compared the gene distribution of these species in the second layer and investigated the sub-terms (e.g. KO in KEGG pathway) if the term had significant different gene numbers in the second layer.

Because many P450 genes were involved in KEGG pathway, we compared P450 genes at the genomic level. All genome sequences were aligned to fungi P450 sequences in Cytochrome P450 database (cut-off e-value: 1e-10 and identity>30%). For glutathione S-transferase (GST) gene, we mapped all genomic genes to GSTs genes described in literature [25], which classified fungal GSTs into different sub families based on phylogenetic analysis (BLASTP cut-off e-value:1e-10 and identity>30%).

### Phylogenetic tree construction and gene families

First, an all-to-all BLASTP alignment was programmed for *G. lucidum* and other 18 species (BLASTP cut-off e-value<1e-7) and core genes which were single-copy in all species were extracted. Then, following a certain order, all core genes of each species were made multiple sequence alignment using the MUSCLE Software and then were concatenated into a super sequence. The Neighbor Joining tree (NJ) was constructed with 1,000 bootstrap replications from aligned sequences by MEGA-5.05 [61]. Based on the all-to-all BLASTP results, Tribe-MCL tools [62] were used to generate the multigene families with default parameters (inflation parameter = 3) in *G. lucidum* and other 7 Agaricomycotina fungi (*F. pinicol, G. trabeum, S. hirstutum, P. chrysosporium, S. commune, L. bicolor*, and *C. cinerea*). The gene families containing genes from only one species were considered as lineage specific. For other gene families (> = 2 members), the evolutionary changes in the protein family size were analyzed using the CAFE program [63], which assesses the protein family expansion or contraction based on the topology of the phylogenetic tree.

### Carbohydrate-related enzymes (CAZymes) and Lignin oxidative enzymes (FOLymes) annotation

As abundant putative genes involved in carbohydrate metabolites and xenobiotics biodegradation in our KEGG analysis, annotations of CAZymes (http://www.cazy.org/) [28] and FOLymes (http://foly.esil.univ-mrs.fr/) [17] were performed using BLASTP analyisis (e-value<1e-10) in *G. lucidum* and other fungi against libraries of CAZy and FOLy database. Refer to [64], a protein was identified as a CAZyme/FOLyme when it showed a similarity score above 50% with sequences of biochemically characterized enzymes. Because FOLyme database is under construction currently, we used genes provided in its publication as seed sequences. In addition, because of the update of database and different parameter chose, the predicted numbers of CAZymes/FOLymes may have a few differences with previous reports.

### Data availability and accession numbers

Data from this Whole Genome Shotgun project have been deposited at DDBJ/EMBL/GenBank (http://www.ncbi.nlm.nih.gov/) under the accession no. AHGX00000000. The version described in this paper is the first version, AHGX01000000. Raw sequencing data have been deposited in the NCBI Sequence Read Archive (http://www.ncbi.nlm.nih.gov/sra) under accession no. SRA048091 and study accession no. SRP009345.

## Supporting Information

File S1 **Table S1. The codon and anti-codon usage of** ***G. lucidum*** **. Table S2.** ***G. lucidum*** **gene annotation. Table S3. Fungi used in the study and their download websites. Table S4. The gene distribution of each fungus in each pathway. Table S5. The number of genes in each gene family and each organism. Table S6.** ***G. lucidum*** **specific gene families as compared to other fungi. Table S7. Protein families showing the highest rate of expansion in** ***G. lucidum*** **genome as compared to other fungi. Table S8. Protein families showing lower size in** ***G. lucidum*** **genome as compared to other fungi. Table S9. The number of genes related vegetative incompatibility in each fungus.**
(XLS)

File S2 **Figure S1. The frequencies of codon usage and anti-codon usage. Figure S2. “Terpenoid backbone biosynthesis” pathway of** ***G. lucidum*** **.**
(DOC)
