# Correction: The Genome of *Ganderma lucidum* Provide Insights into Triterpense Biosynthesis and Wood Degradation

**DOI:** 10.1371/annotation/9f521140-7406-425a-bc90-d0f6075dc854

**Published:** 2012-05-08

**Authors:** Dongbo Liu, Jing Gong, Wenkui Dai, Xincong Kang, Zhuo Huang, Hong-Mei Zhang, Wei Liu, Le Liu, Junping Ma, Zhilan Xia, Yuxin Chen, Yuewen Chen, Depeng Wang, Peixiang Ni, An-Yuan Guo, Xingyao Xiong

There were typographical errors in the article title and citation. The correct title is: The genome of *Ganoderma lucidum* provides insights into triterpenes biosynthesis and wood degradation. The correct citation is: Liu D, Gong J, Dai W, Kang X, Huang Z, et al. (2012) The genome of *Ganoderma lucidum* provides insights into triterpenes biosynthesis and wood degradation. PLoS ONE 7(5): e36146. doi:10.1371/journal.pone.0036146 

