# Supplementary material for: The Genome of Ganderma lucidum Provide Insights into Triterpense Biosynthesis and Wood Degradation
Source: PLoS One. 2012 May 2;7(5):e36146. doi: 10.1371/journal.pone.0036146 (PMC3342255; doi:10.1371/journal.pone.0036146)
Supplement: File S2 — Figure S1. The frequencies of codon usage and anti-codon usage. Figure S2. “Terpenoid backbone biosynthesis” pathway of G. lucidum . (DOC) [file pone.0036146.s002.doc]

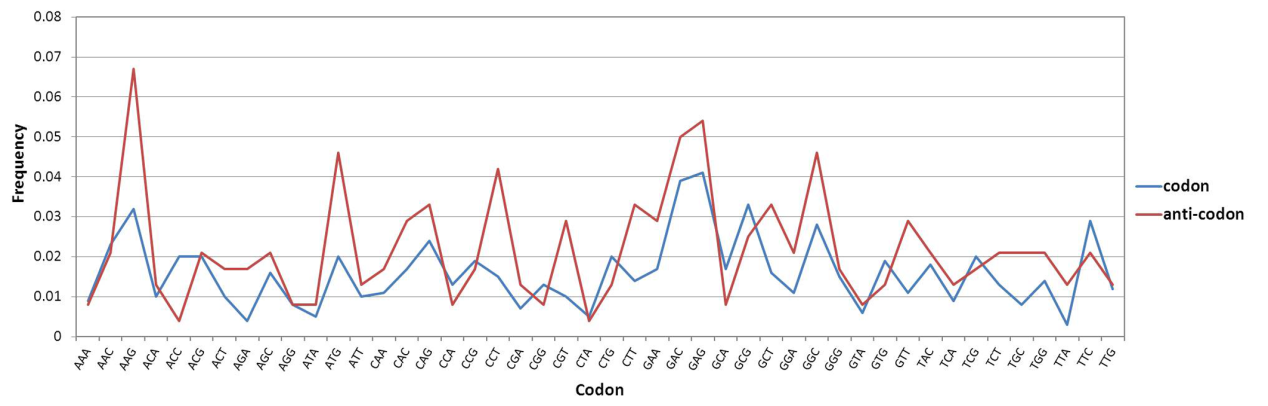


**Figure S1. The frequencies of codon usage and anti-codon usage in *G. lucidum*.**


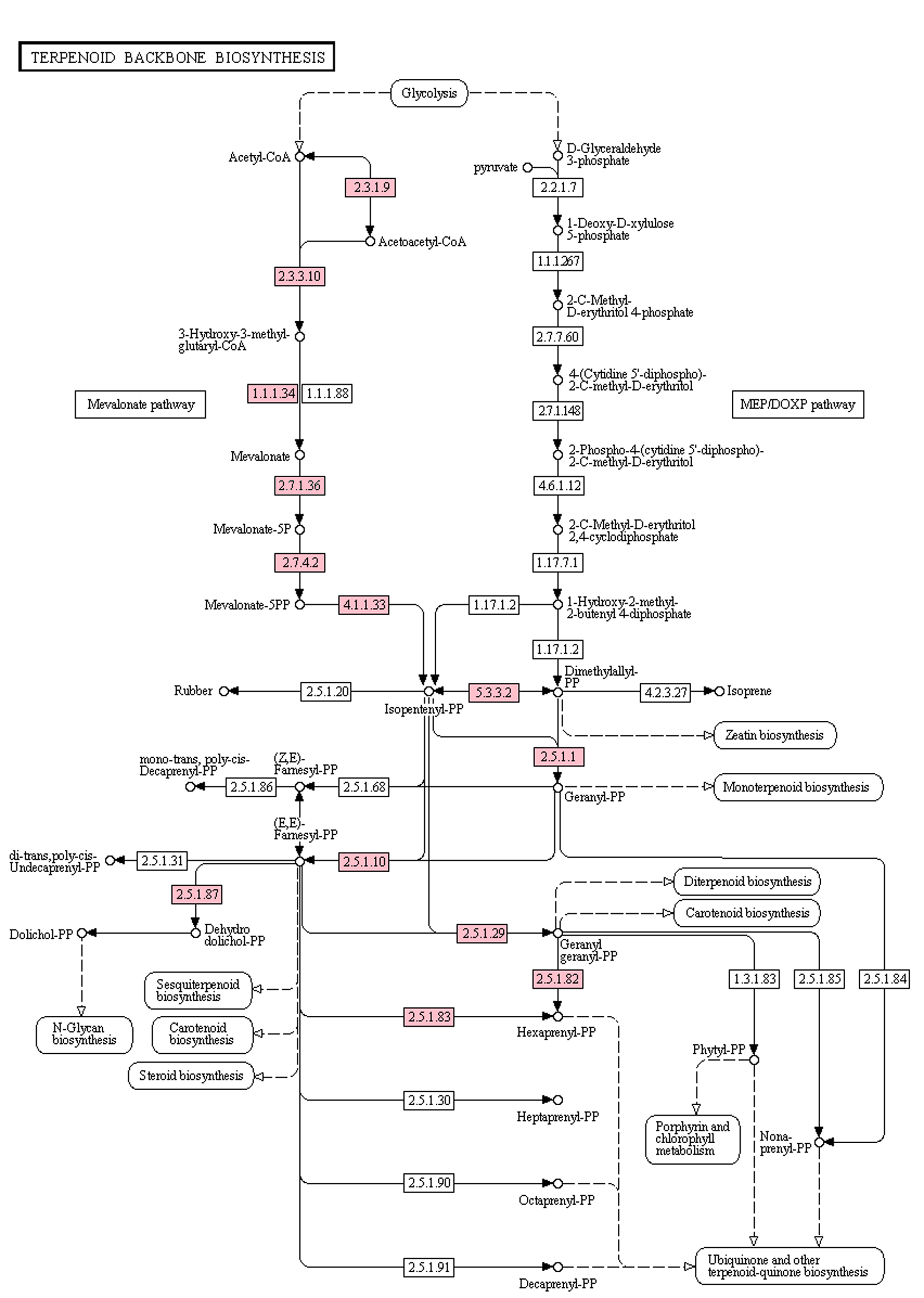


**Figure S2. “Terpenoid backbone biosynthesis” pathway of** ***G. lucidum.*** The red box indicates existing homologous genes of the enzyme, while white box means not. The photo was done by KEGG mapper.
